# Supplementary material for: The impact of roadway conditions towards accident severity on federal roads in Malaysia
Source: PLoS One. 2020 Jul 6;15(7):e0235564. doi: 10.1371/journal.pone.0235564 (PMC7337329; doi:10.1371/journal.pone.0235564)
Supplement: Attachment — Submitted filename: Response to Reviewers comments.docx [file pone.0235564.s002.docx]

**Subject**: Response to reviewer’s comments

**Manuscript Number**: PONE-D-20-07171

**Manuscript Title:** THE IMPACT OF ROADWAY CONDITIONS TOWARDS ACCIDENT SEVERITY ON FEDERAL ROADS IN MALAYSIA

Dear Professor

Feng Chen, Ph.D.

Academic Editor

PLOS ONE

Thank you very much for your efforts in handling our manuscript. We hereby acknowledge the positive comments and considerations received from reviewers and editor to improve the contents of our manuscript. We gratefully think that the comments have helped us to further enhance and strengthen the paper. The required amendments have been performed and the revised sections were highlighted throughout the manuscript. We hope that the revised manuscript is now acceptable for publication in your journal. Please find below our response/rebuttal to the reviewers’ comments.

The revised manuscript is submitted for your positive consideration and acceptance.

Yours sincerely

Sitti Asmah Hassan, PhD

[sasmah@utm.my](mailto:sasmah@utm.my)

|  | **COMMENTS** | **ANSWER** |
| --- | --- | --- |
| 1. | We note that Figure 1 in your submission contain map/satellite images which may be copyrighted. All PLOS content is published under the Creative Commons Attribution License (CC BY 4.0), which means that the manuscript, images, and Supporting Information files will be freely available online, and any third party is permitted to access, download, copy, distribute, and use these materials in any way, even commercially, with proper attribution. For these reasons, we cannot publish previously copyrighted maps or satellite images created using proprietary data, such as Google software (Google Maps, Street View, and Earth). For more information, see our copyright guidelines: http://journals.plos.org/plosone/s/licenses-and-copyright.  We require you to either (1) present written permission from the copyright holder to publish these figures specifically under the CC BY 4.0 license, or (2) remove the figures from your  submission: | 1. Figure 1 was drawn by authors based on data reported by the refer Malaysian Automotive Association. Malaysia Automotive Info “Summary of Sales & Production   Data.” Malaysia; 2017 (reference no. 10). Citation was added to the caption of the figure.   2. I believe the comment was made for Figure 2. Figure 2 contains map image. The figure was removed from the manuscript. Caption of Figure 2 is as below:   Fig 2. Federal roads involve in this particular study was indicated in red line (Source: Public Work Department) |
|  | 1. You may seek permission from the original copyright holder of Figure 1 to publish the content specifically under the CC BY 4.0 license.  We recommend that you contact the original copyright holder with the Content Permission Form (http://journals.plos.org/plosone/s/file?id=7c09/content-permission-form.pdf) and the  following text:  “I request permission for the open-access journal PLOS ONE to publish XXX under the Creative Commons Attribution License (CCAL) CC BY 4.0 (http://creativecommons.org/licenses/by/4.0/).  Please be aware that this license allows unrestricted use and distribution, even commercially, by third parties. Please reply and provide explicit written permission to publish XXX under a CC BY license and complete the attached form.”  Please upload the completed Content Permission Form or other proof of granted permissions as an "Other" file with your submission.  In the figure caption of the copyrighted figure, please include the following text: “Reprinted from [ref] under a CC BY license, with permission from [name of publisher], original copyright  [original copyright year].”  2. If you are unable to obtain permission from the original copyright holder to publish these figures under the CC BY 4.0 license or if the copyright holder’s requirements are incompatible with the CC BY 4.0 license, please either i) remove the figure or ii) supply a replacement figure that complies with the CC BY 4.0 license. Please check copyright information on all replacement  figures and update the figure caption with source information. If applicable, please specify in the figure caption text when a figure is similar but not identical to the original image and is  therefore for illustrative purposes only.  The following resources for replacing copyrighted map figures may be helpful:  USGS National Map Viewer (public domain): http://viewer.nationalmap.gov/viewer/  The Gateway to Astronaut Photography of Earth (public domain): http://eol.jsc.nasa.gov/sseop/clickmap/  Maps at the CIA (public domain): https://www.cia.gov/library/publications/the-world-factbook/index.html and https://www.cia.gov/library/publications/cia-maps-publications/index.html  NASA Earth Observatory (public domain): http://earthobservatory.nasa.gov/  Landsat: http://landsat.visibleearth.nasa.gov/  USGS EROS (Earth Resources Observatory and Science (EROS) Center) (public domain): http://eros.usgs.gov/#  Natural Earth (public domain): http://www.naturalearthdata.com/ |  |
|  | **REVIEWER COMMENTS TO THE AUTHORS:** | **RESPONSE** |
| 2. | **Reviewer 1:**  This paper investigates the effects of roadway conditions on traffic crash severity on Federal roads in Malaysia. Although the authors have found something, the contributions of the findings are very limited. There are several reasons for the limitations of this research: | **Response to Reviewer 1:** |
|  | 1. Only the fatal crash data are used in the analysis which cannot provide a comprehensive understanding of crash severity (usually including other severity levels such as no injury, minor injury, and severe injury). Focusing on fatal crashes also significantly reduces the size of crash dataset, which may result in small size problem on model parameter estimation. | 1. Thanks for your kind observation, critics and comments in the overall improvement of the manuscript.   Road accident fatalities have become top priorities and concerns for Malaysia policymakers, hence, understanding the principal factors that explain accident fatality is considered to be the first step towards the adequate design of an accident prevention strategy related to road condition. This was the original motivation in the previous analysis.  Based on the comments received, the new data analysis was performed by including all accident severity (no injury, slight injury, serious injury and fatal injury).  We admit the small sample size issues. However, the raw data obtained was from Royal Malaysia Police and Public Work Department database (from 2008 to 2015). |
|  | 2. The binary Logistic regression is too simple, which cannot guarantee the precise of the estimation results. Please refer to the Mannering and Bhat (2014) and Savolainen et al. (2011) for the introduction of more advanced methods. | 2. The initial motivation of the study was to adopt the logistic regression approach based on the availability & suitability of data source/type (Reference: Al-Ghamdi AS. Using logistic regression to estimate the influence of accident factors on accident severity. Accident Analysis and Prevention. 2002;34(6):729–741.).   Based on your suggestion, we re-evaluate the propose model in the revised manuscript by utilizing Ordered Logit Model (OLM)- Mannering and Bhat (2014) and Savolainen et al. (2011). Additional related references were also included. |
|  | 3. The factors related to drivers, vehicles, and weather conditions which have been found to significantly impact crash severity, are not controlled in the analysis, which may also lead to biased inference. At least, major revisions are required; otherwise, I do not recommend its publication. | 3. We admit that the accident may due to several factors. The data collection was obtained from a traditional data source in which it is limited in many ways. The Royal Malaysia Police and Public Work Department (PWD) database revealed the accident severity and variables associated to it mostly on road conditions. For instance, the roadway design and maintenance is under the job scope of traffic engineer. Therefore, explanatory variables related to roadway conditions is worth to be explored in Malaysia condition for safety mitigation strategies at practitioners’ levels. |
|  | Some other comments are as follows:  As the current research focuses on analyzing crash severity, the review part of methods for crash frequency should be removed from the Introduction section. Instead, more introduction on the crash severity models should be added.  According to information in the data collection section, there are about 900 serious or minor injury crashes collected. Why are not they used in the analysis of crash severity?  In Table 1, the definitions of good and bad conditions of each variable should be described. They are very vague to readers.  In Table 3, the authors seems to copy the results from the SPSS immediately. The terms, such as “B” and “S.E.”, are not formal.  Mannering, F. L., Bhat C. R., 2014. Analytic methods in accident research: Methodological frontier and future directions. Analytic Methods in Accident Research 1: 1-22.  Savolainen, P. T., Mannering, F. L., Lord, D., Quddus, M. A., 2011. The statistical analysis of highway crash-injury severities: A review and assessment of methodological alternatives. Accident  Analysis and Prevention 43 (5), 1666-1676. | - Methods for crash frequency models have been removed from the manuscript. - Alternatively, crash severity models have been included in the revised manuscript. - Understanding the fatal contributing factors was the initial motivation of the study. However, based on your recommendation, the revised version was made to include all accident severity. - This statement was made before the appearance of Table 1: “The good and poor characteristics of these variables were classified when they conformed to the PWD specification and not in conformity, respectively.” Reference related to the PWD specification was also added at the end of the statement in the revised manuscript. - “B” and “SE” were omitted from the manuscript. |
| 3. | **Reviewer 2:**  This paper claims to investigate the impact of roadway conditions on traffic accident severity of federal road in Malaysia. There are some serious flaws with the manuscript as  listed below:  1. The data adopted in this paper only included 166 accidents with 1067 cases. This raises several concerns about the validity of the paper. First of all, an investigation towards injury severity  using only 166 accidents suffers greatly from the small sample size issues. By processing 166 accidents into 1067 cases makes it even worse. Since it means one accident is split into multiple  cases where the severity outcomes will be different but the nine explanatory variables remains the same.  2. The simple logistic regression is not appropriate for analyzing injury severity because it doesn't not account for unobserved heterogeneity issues often present in crash data. | **Response to Reviewer 2:**   1. Thanks for your kind observation, critics and comments. We have provided the answer of the same issue mentioned by reviewer #1.   Road accident fatalities have become top priorities and concerns for Malaysia policymakers, hence, understanding the principal factors that explain accident fatality is considered to be the first step towards the adequate design of an accident prevention strategy related to road condition. This was the original motivation in the previous analysis. We admit the small sample size issues, but this was the original data obtained from Royal Police Malaysia and Public Work Department database.  Given the limitation in data, accident severity model in this study can be used at practitioners’ level in which all the explanatory variables are within their authority.  Due to small data size, we are unable to consider unobserved heterogeneity factors. According to Mannering et al., 2016, random parameters, latent class, and other unobserved heterogeneity approaches will mitigate the adverse impacts of omitting significant explanatory variables, the resulting model estimates will not be able to track the unobserved heterogeneity as well as when having the significant omitted variables included in the specification. **Thus leaving out important explanatory variables still remains a problem even with advanced approaches to account for unobserved heterogeneity.**  However, statistical approaches that address unobserved heterogeneity tend to be more complex from a model estimation perspective (Reference: Mannering, F. L., Shankar, V., & Bhat, C. R. (2016). Unobserved heterogeneity and the statistical analysis of highway accident data. *Analytic Methods in Accident Research*, *11*, 1–16)     2. Based on your input, the initial binary logistic regression model is now replaced by ordered logistic regression model. All accident severities were evaluated in the new analysis (no injury, slight injury, serious injury, and fatal). |
