# Supplementary material for: The impact of roadway conditions towards accident severity on federal roads in Malaysia
Source: PLoS One. 2020 Jul 6;15(7):e0235564. doi: 10.1371/journal.pone.0235564 (PMC7337329; doi:10.1371/journal.pone.0235564)
Supplement: Attachment — Submitted filename: Response to Reviewers comments.docx [file pone.0235564.s003.docx]

**Subject**: Response to reviewer’s comments

**Manuscript Number**: PONE-D-20-07171R1

**Manuscript Title:** THE IMPACT OF ROADWAY CONDITIONS TOWARDS ACCIDENT SEVERITY ON FEDERAL ROADS IN MALAYSIA

Dear Professor

Feng Chen, Ph.D.

Academic Editor

PLOS ONE

Thank you very much for your efforts in handling our manuscript. We hereby acknowledge the positive comments and considerations received from reviewers and editor to improve the contents of our manuscript. We gratefully think that the comments have helped us to further enhance and strengthen the paper. The required amendments have been performed and the revised sections were highlighted throughout the manuscript. We hope that the revised manuscript is now acceptable for publication in your journal. Please find below our response/rebuttal to the reviewers’ comments.

The revised manuscript is submitted for your positive consideration and acceptance.

Yours sincerely

Sitti Asmah Hassan, PhD

[sasmah@utm.my](mailto:sasmah@utm.my)

|  | **REVIEWER COMMENTS TO THE AUTHORS:** | **RESPONSE** |
| --- | --- | --- |
| 1. | **Reviewer #1:**  Reviewer #1: The authors should be thanked for their great efforts on improving the manuscript. Most of my comments have been addressed properly. A minor suggestion is that more works on generalized ordered logit models should be acknowledged in the Introduction Section, including:  Investigating the impacts of real-time weather conditions on freeway crash severity: A Bayesian spatial analysis. International Journal of Environmental Research and Public Health, 2020, 17(8), 2768.  Analyzing freeway crash severity using a Bayesian spatial generalized ordered logit model with conditional autoregressive priors. Accident Analysis and Prevention, 2019, 127, 87-95. | **Response to Reviewer #1:**  Thanks for your suggestions.  The additional works on the GLOM have been added in the Introduction section. |
| 2. | **Reviewer #2:**  The paper has addressed most of the concerns about the paper, however, the following relevant papers should be discussed in the literature review and included in the reference:  [1] Feng Chen, Mingtao Song and Xiaoxiang Ma, Investigation on the Injury Severity of Drivers in Rear-End Collisions Between Cars Using a Random Parameters Bivariate Ordered Probit Model, International Journal of Environmental Research and Public Health, 2019, 16(14) , 2632.  [2] Chen, Feng; Chen, Suren; Ma, Xiaoxiang. Analysis of hourly crash likelihood using unbalanced panel data mixed logit model and real-time driving environmental big data. 2018, JOURNAL OF SAFETY RESEARCH. 65: 153-159.  [3] Bowen Dong, Xiaoxiang Ma, Feng Chen and Suren Chen. “Investigating the Differences of Single- and Multi-vehicle Accident Probability Using Mixed Logit Model", Journal of Advanced Transportation, 2018, UNSP 2702360.  [4] Ma, X., Chen, F., Chen, S., 2015. Empirical analysis of crash injury severity on mountainous and non-mountainous interstate  highways. Traffic Inj. Prev. 16 7 , 715–723. doi:10.1080/15389588.2015.1010721 | **Response to Reviewer #2:**  Thanks for your suggestions.  The additional references have been added in the manuscript. |
